# Chronotype preference and glycemic control in type 2 diabetes

**DOI:** 10.1093/sleep/zsab195

**Published:** 2021-08-16

**Authors:** Pei Xue, Xiao Tan, Xiangdong Tang, Christian Benedict

**Affiliations:** 1 Department of Neuroscience (Sleep Science, BMC), Uppsala University, Uppsala, Sweden; 2 Sleep Medicine Center, Department of Respiratory and Critical Care Medicine, Mental Health Center, Translational Neuroscience Center, and State Key Laboratory of Biotherapy, West China Hospital, Sichuan University, Chengdu, China

Chronotype, i.e. an individual’s preference for morningness or eveningness, has been linked to type 2 diabetes. For example, in the Nurses’ Health Study 2 following 64 615 women from 2005 to 2011, evening preference among day workers was associated with a higher type 2 diabetes risk [1]. Chronotype may also influence glycemic control among those already treated for type 2 diabetes. Using questionnaire data from 194 patients with type 2 diabetes, it has, for example, been shown that those with a later midpoint of sleep exhibited higher hemoglobin A1c (HbA1c) values compared with patients with an earlier midpoint of sleep [2]. The HbA1c is a widely used clinical marker for assessing long-term glycemic control [3].

While previous studies have mainly focused on its association with the type 2 diabetes risk and HbA1c, the possible impact of chronotype on other key clinical markers of type 2 diabetes is less well studied. For example, according to the American Diabetes Association, a key treatment target for type 2 diabetes is an HbA1c of less than 7.0% [4]. Additionally, insulin treatment is recommended in patients with type 2 diabetes exhibiting a secondary failure to first-line oral antidiabetic treatment [5]. Given previous reports suggesting that later chronotype impairs glycemic control [2], it could be hypothesized that patients with type 2 diabetes reporting eveningness preference would exhibit lower odds of having an HbA1c of less than 7.0% and increased odds of being treated with insulin compared with those reporting either intermediate or morningness preference. To test these hypotheses, in the present study, we used data from 11 594 patients with type 2 diabetes who participated in the UK Biobank baseline investigation between 2006 and 2010.

A case of type 2 diabetes was considered confirmed if at least one of the following was reported: (1) by an algorithm based on self-reported disease, medication, and type 2 diabetes diagnosis in medical history [6]; and (2) HbA1c greater than 6.5% (measured by Bio-Rad VARIANT II TURBO HbA1c analyzer, Bio-Rad, Hercules, CA) and the use of hypoglycemic medications (e.g. metformin). Data on subjects’ antidiabetic pharmacotherapy were taken from the UK Biobank verbal interview. Insulin therapy was prescribed either as a monotherapy or component of a combination therapy.

In total, we identified 16 945 patients treated for type 2 diabetes. Patients were excluded from analysis due to missing data on chronotype (*N* = 2128), HbA1c (*N* = 1051), and confounders (for description, see below; *N* = 1981). For the analysis, we also removed cases with HbA1c *z*-values greater than 3 and smaller than −3 (*N* = 191). Thus, 11 594 patients with type 2 diabetes remained available for analysis. Circadian preference was assessed by the question “Do you consider yourself to be?” with one of six possible answers: definitely morning, more morning than evening, more evening than morning, and definitely evening. Do not know or prefer not to answer responses were coded as missing. The UK Biobank received ethics approval from the National Health Service Research Ethics Service (reference 11/NW/0382).

To test the associations of chronotype with the odds of having an HbA1c of less than 7% (52.4% of the cohort) and being on insulin treatment (20.4% of the cohort), we performed logistic regression analyses (SPSS 24.0, Inc., Chicago, IL). The results are presented as unadjusted or adjusted odds rations with 95% confidence intervals. The following confounders were considered in the adjusted analysis: participants’ age at UK Biobank investigation, sex, Townsend index reflecting socioeconomic status, body mass index, ethnicity, hypertension status (defined as systolic blood pressure ≥140 mmHg), smoking status, level of physical activity (divided into low to moderate vs high level, according to the short-form International Physical Activity Questionnaire (IPAQ) based on the total metabolic equivalent minutes per week), sleep duration, snoring (partner or a close relative or friend complained about UK Biobank participants’ snoring), difficulty falling and staying asleep (if the patient responded with “usually” to the question: Do you have trouble falling asleep at night or do you wake up in the middle of the night?), age at diagnosis of type 2 diabetes, the therapeutic regime of type 2 diabetes (including medications such as metformin, glitazone, sulfonylurea, meglitinide, and insulin), and region of the assessment center. In all logistic regression analyses, patients reporting definitely morning circadian preference were set as the reference group.

**Figure 1.**
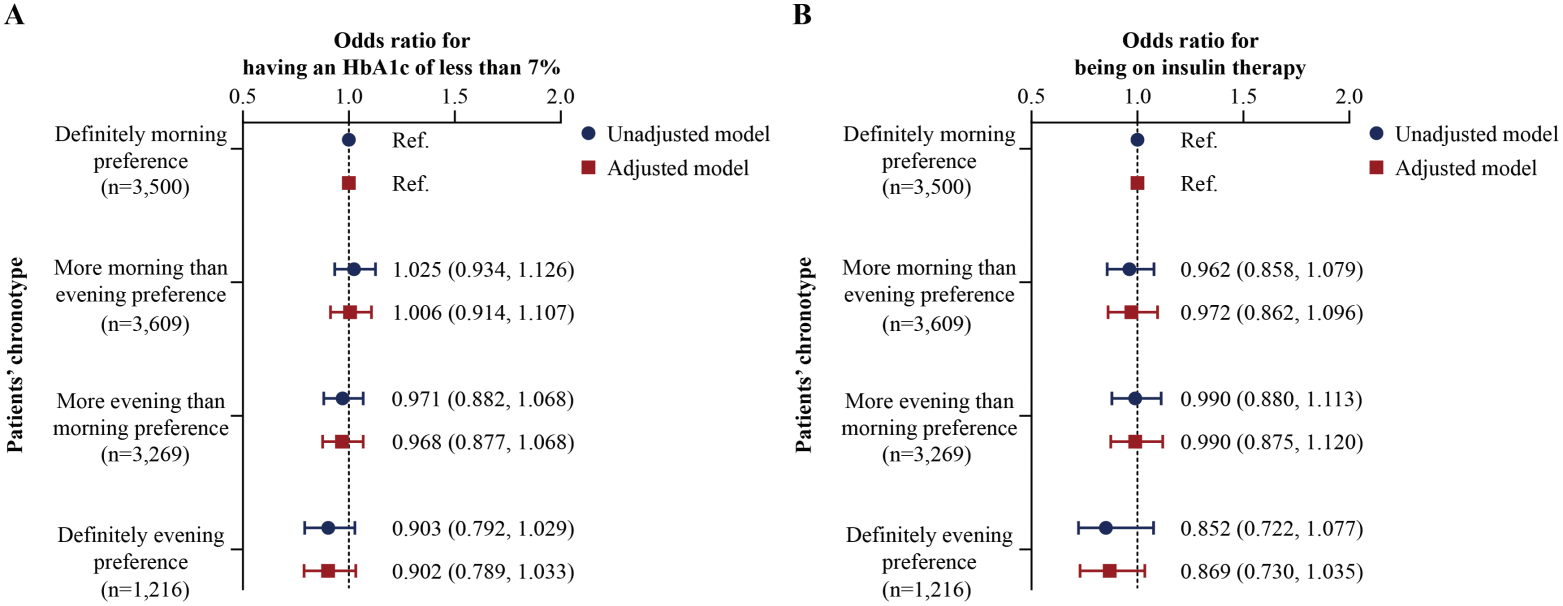
Unadjusted and adjusted odds ratios and 95% CIs for (A) having an HbA1c of less than 7% and (B) being on insulin therapy, stratified by patients’ chronotype. Logistic regression was adjusted for participants’ age at UK Biobank investigation, sex, Townsend index, body mass index, ethnicity, systolic hypertension, smoking status, level of physical activity, sleep duration, snoring, difficulty falling and staying asleep, age at diagnosis of type 2 diabetes, antidiabetic drug regime, and region of the assessment center. Abbreviation: CIs, confidence intervals.

A univariate analysis of variance revealed that the HbA1c varied by circadian preference (*p* = 0.015); however, the differences were small and may therefore be less clinically important (definitely morning circadian preference: 7.1 ± 1.1%; more morning than evening circadian preference: 7.1 ± 1.1%; more evening than morning circadian preference: 7.1 ± 1.1%; and definitely evening circadian preference: 7.2 ± 1.1%). As shown by both unadjusted and adjusted logistic regression analyses, no statistically significant association of chronotype with the therapeutic goal of having an HbA1c of less than 7% was found (Figure 1, A). Likewise, chronotype did not significantly alter the odds ratio of being treated with insulin (Figure 1, B).

The present cross-sectional study represents one of the largest investigations to date into the possible association of chronotype with glycemic control among patients with type 2 diabetes. If confirmed by future studies, our results suggest that key clinical markers of long-term glycemic control do not differ in a clinically relevant manner by chronotype among patients with type 2 diabetes. However, in interpreting these findings, it is important to consider that a single question was used to assess chronotype. Importantly, this question overlaps with the final question of the Morningness-Eveningness Questionnaire [7], which is a strong correlate of circadian preference [7]. It must also be borne in mind that circadian variables not measured herein, such as social jetlag—defined as the time difference between the midpoint of sleep on workdays and on free days [8]—and sleep duration regularity, may play a more significant role than chronotype for glycemic control among patients with type 2 diabetes. For example, in a study involving 252 type 2 diabetes patients, social jetlag but not chronotype was a significant predictor of HbA1c levels [9]. In a separate study with 172 patients with type 2 diabetes, it was further shown that larger variability in sleep duration assessed by wrist-actigraphy and sleep questionnaires were strongly associated with HbA1c [10].
